# Effectiveness of herb-partitioned moxibustion on the navel for pregnancy outcomes in patients with recurrent implantation failure undergoing in vitro fertilization and embryo transfer: a study protocol for a randomized controlled trial

**DOI:** 10.1186/s13063-022-06156-5

**Published:** 2022-03-15

**Authors:** Qingchang Xia, Shuzhong Gao, Jingyan Song, Dongqing Du, Chunjing Li, Yue Zhou, Xiaobin Zhang, Zhibin Dong, Yuning Ma, Yuxia Ma

**Affiliations:** 1 College of Acupuncture and Massage, Shandong University of Traditional Chinese Medicine, Jinan, 250014 China; 2 Reproductive and Genetic Center of Integrated Traditional and Western Medicine, The Affiliated Hospital of Shandong University of Traditional Chinese Medicine, Jinan City, 250011 Shandong Province China

**Keywords:** Herb-partitioned moxibustion on the navel, Frozen embryo transfer, Recurrent implantation failure, Traditional Chinese medicine

## Abstract

**Background:**

Recurrent implantation failure (RIF) in the majority of patients undergoing in vitro fertilization and embryo transfer (IVF-ET) is caused by various factors such as maternal age, embryo quality, endometrial receptivity (ER), and immunity. The incidence of RIF is usually between 5 and 10%. Previous studies have shown that herb-partitioned moxibustion on the navel is one of the treatment methods of acupuncture with a positive effect on pregnancy. However, its application in the treatment of RIF has not been reported. Therefore, this study aims to evaluate the effectiveness and safeness of herb-partitioned moxibustion on the navel in improving the outcome of frozen embryo transfer (FET) in patients with RIF.

**Methods:**

This study conducts a randomized controlled trial (RCT). It is planned to recruit 210 patients undergoing RIF for FET from Affiliated Hospital of Shandong University of Traditional Chinese Medicine and randomly divide them into the treatment group and the control group in a ratio of 1:1. The patient of the treatment group will be treated with herb-partitioned moxibustion on the navel once a week for three consecutive menstrual cycles. No intervention will be used in the control group for 3 menstrual cycles. In the fourth menstrual cycle, all patients will undergo artificial cycle to prepare the endometrium for FET. The pregnancy outcomes will be recorded after a 3-month follow-up. Primary outcome will be assessed as the ongoing pregnancy rate compared with the control group. Secondary outcomes include the endometrial type, resistance index (RI), pulsatility index (PI) of the bilateral uterine artery, endometrial blood flow, serum estradiol (E_2_), progesterone (P), biochemical pregnancy rate, implantation rate, and clinical pregnancy rate.

**Discussion:**

If the results show that the herb-partitioned moxibustion on the navel can improve IVF-ET outcomes in patients with RIF, it will be recommended in clinical practice.

**Trial registration:**

Chinese Clinical Trial Registry (ChiCTR) ChiCTR2100043954. Registered on 8 July 2021.

**Supplementary Information:**

The online version contains supplementary material available at 10.1186/s13063-022-06156-5.

## Introduction

### Background and rationale

Since the birth of the first test-tube baby in 1978, the technology of IVF-ET has been continuously innovated and developed. This innovation has made significant improvements in conception rate, implantation rate, and live birth rate. However, due to factors such as maternal age [1, 2], embryo quality [3], ER [4, 5], and immunity [6, 7], some patients who have received high-quality embryo transfers had unsuccessful pregnancy whereas more than 50% of the patients ended up with implant failure or biochemical abortion [8]. By definition, RIF is when women under the age of 40 years fail to achieve a clinical pregnancy after transplantation of at least four high-quality embryos in at least three fresh or frozen cycles [9, 10]. It has been reported that the incidence of RIF in IVF-ET patients may reach 5–10% [11], so RIF has become an issue of concern in current reproductive research.

**Fig. 1:**
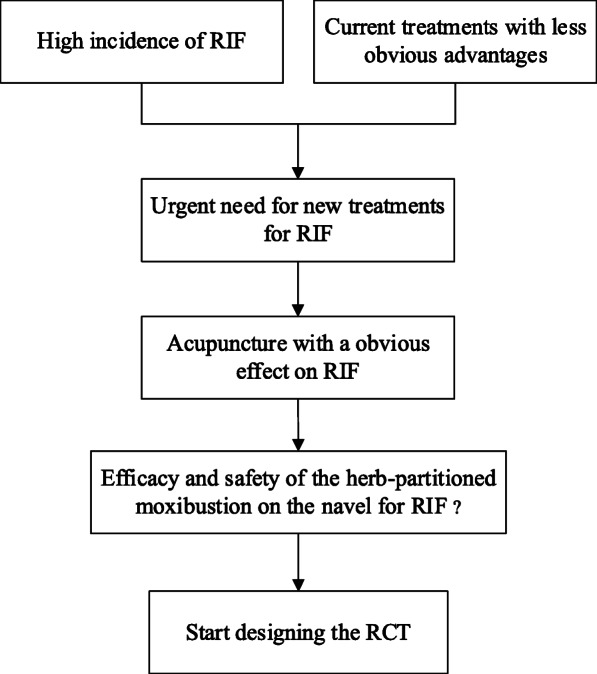
Research background flowchart

In Western medicine, the main treatments for RIF are surgery and drugs. Surgical treatment includes hysteroscopic operation before transplantation to stimulate endometrial repair and growth by scraping the endometrium [12–14]. On the other hand, drug treatment mainly includes human chorionic gonadotropin [15], growth hormone [16], glucocorticoid [17], and oral letrozole [18]. Western medicine is adopted when RIF is combined with other diseases, due to the lack of effective treatment measures for RIF alone [19]. As a characteristic and representative way of traditional medicine, acupuncture has a comprehensive treatment effect of multiple channels, multiple links, and multiple targets. It treats systemic diseases with specific operation methods and conduction of meridians and acupoints. It has the characteristic that “internal disease is treated by external intervention” [20]. Therefore, it has been gradually accepted by domestic and foreign experts in the field of reproduction and is also applied in the prevention and treatment of RIF. The results have suggested that acupuncture has a significant effect on improving the implantation rate and clinical pregnancy rate in patients undergoing RIF [21, 22]. Herb-partitioned moxibustion on the navel belongs to the category of acupuncture. This method uses the Shenque acupoint (CV8) as the operating position. It puts medicinal powder in Shenque acupoint and cooperates with moxibustion to stimulate meridian qi. Further, it dredges meridian-collaterals, regulates qi-blood and functions of internal organs, and prevents and cures diseases. The structure of the Shenque acupoint is the most special among all acupuncture points. According to a previous study [23], it has been confirmed that it has a clear vascular structure from the perspective of vascular biology. Moxibustion can directly act on the intima and promote its microcirculation, which is the basis of Shenque treatment effect specificity. In addition, it has been found that in infertility patients with hypoluteal function, herb-partitioned moxibustion on the navel can effectively reduce RI and PI, and then improve the intrauterine blood perfusion, increase the endometrial thickness, and ultimately improve the pregnancy rate [24]. Another study [25] have found that herb-partitioned moxibustion on the navel can improve the quality of oocytes in elderly infertile patients by regulating the retinol metabolism pathway and glycerophospholipid metabolism pathway. However, there are no cases of herb-partitioned moxibustion on the navel for the treatment of RIF. Therefore, this study intended to use an RCT to observe the therapeutic effect of herb-partitioned moxibustion on the navel for RIF and explore its mechanism. In conclusion, Fig. 1 summarizes the preceding background to the study flowchart.

## Methods

### Study objective

This study aims to investigate the effect of herb-partitioned moxibustion on the navel on the improvement of the outcomes of FET in patients with RIF.

### Trial design and setting

**Fig. 2:**
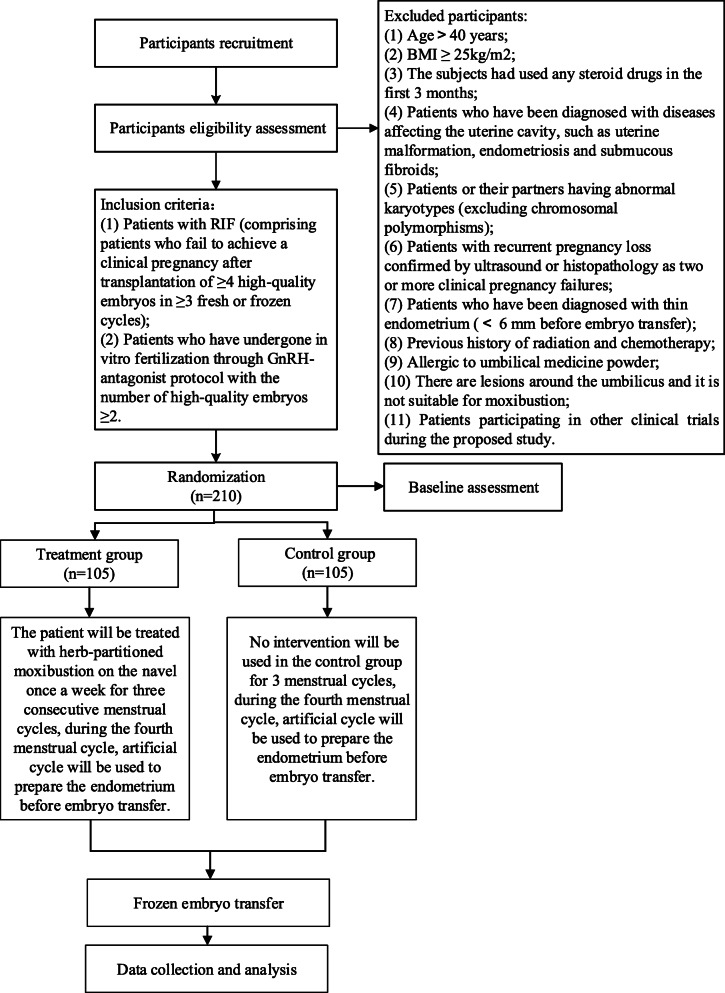
The study flowchart

This is an RCT study. Participants will be recruited from the Affiliated Hospital of Shandong University of Traditional Chinese Medicine. Eligible participants will be randomly divided into the treatment (herb-partitioned moxibustion on the navel) or control group at a ratio of 1:1. The study flow chart and schedule are as shown in Fig. 2 and Table 1 (SPIRIT figure) respectively. The SPIRIT checklist is presented in Additional file 1. The main study sites will be the center of reproduction and genetics of Integrated Chinese and Western Medicine and the Center of Traditional Chinese Medicine External Treatment in the Affiliated Hospital of Shandong University of Traditional Chinese Medicine. This protocol follows the SPIRIT Reporting Guidelines [26].

**Table 1:** Schedule of the study process (SPIRIT figure)

	Study period						
	Enrollment	Post allocation					
Timepoint	1st visit	1st MC	2nd MC	3rd MC	4th MC	14 days after FET	3 months after FET
**Enrollment:**							
Eligibility screen	√						
Informed consent	√						
Allocation	√						
**Interventions:**							
Herb-partitioned moxibustion on the navel		√	√	√			
Artificial cycle					√		
**Assessments:**							
Serum estrogen					√	√	√
Serum progesterone					√	√	√
Ultrasonography	√	√	√	√	√	√	√
Pregnancy test					√	√	
Pregnancy outcome					√	√	√
Telephone follow-up						√	√

### Eligibility criteria

#### Inclusion criteria

Patients who meet the following criteria will be enrolled in this study:

(1) Patients with RIF (comprising patients who fail to achieve a clinical pregnancy after transplantation of ≥ 4 high-quality embryos in ≥ 3 fresh or frozen cycles [11]) and (2) patients who have undergone in vitro fertilization through GnRH-antagonist protocol with the number of high-quality embryos ≥ 2.

#### Exclusion criteria

(1) Age > 40 years; (2) BMI ≥ 25 kg/m 2[27]; (3) the subjects had used any steroid drugs in the first 3 months; (4) patients who have been diagnosed with diseases affecting the uterine cavity, such as uterine malformation, endometriosis, and submucous fibroids; (5) patients or their partners having abnormal karyotypes (excluding chromosomal polymorphisms); (6) patients with recurrent pregnancy loss confirmed by ultrasound or histopathology as two or more clinical pregnancy failures; (7) patients who have been diagnosed with thin endometrium ( < 6 mm before embryo transfer); (8) previous history of radiation and chemotherapy; (9) allergic to umbilical medicine powder; (10) there are lesions around the umbilicus and it is not suitable for moxibustion; and (11) patients participating in other clinical trials during the proposed study.

### Dropout criteria

Patients who voluntarily withdraw or have poor clinical compliance during the observation period or are found not to meet the inclusion criteria after enrolment will be considered as having dropped out.

### Interventions

The treatments will be performed by a licensed acupuncturist who has at least 5 years of experience with acupuncture.

### Treatment group

Patients in the treatment group will be administered with herb-partitioned moxibustion on the navel once a week for three consecutive menstrual cycles. The artificial cycle will be used in endometrium preparation during the fourth menstrual cycle before embryo transfer.

**Fig. 3:**
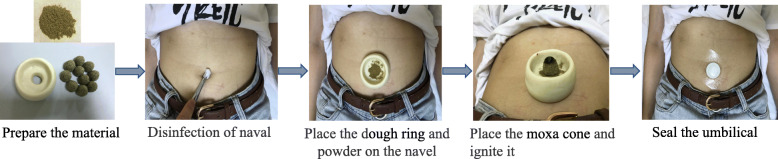
The operation process of the herb-partitioned moxibustion on the navel

The specific operations of herb-partitioned moxibustion on the navel are as follows [28]: (1) preparation powder of herb-partitioned moxibustion on the navel: Loranthus parasiticus, Semen cuscutae, Eucommia ulmoides, Cinnamon, Peach kernel, Red peony root, Angelica sinensis, Ligusticum chuanxiong, Rhizoma cyperi, and Borneol will be mixed in a certain proportion and crushed through the micro sifter and stored them in a dark tank for later use. (2) To prepare a dough ring: Make the flour into a ring with warm boiling water for later use. The inner diameter of the ring is about 3 cm, the outer diameter is about 6 cm, the inner wall height is about 2 cm, and the outer wall height is about 3 cm. The middle hole is the same size as the patient's umbilical hole. (3) Preparation of moxa cone: Moxa is squeezed into a cone-shaped body (about 1 cm in diameter and 1 cm in height) with a flat bottom and a tip and it is required to be twisted tightly and set aside. (4) Operation: The patients are placed in the supine position, and the dough ring is placed over the navel after routine disinfection of the umbilicus with 75% ethanol, the middle hole of the dough ring corresponding to the navel, and then take the powder of herb (about 8–10 g) and fill the belly button with moderate tension. Then, put the moxa cone over the powder, and add a new one after burning it out naturally. The procedure will take approximately 2 h and approximately 10 moxa cones will be used. Patients will be asked to close their eyes and rest during the procedure. After moxibustion, the umbilicus will be affixed and sealed with a medical applicator and take it off after 24 h. The operation process of the herb-partitioned moxibustion is presented in Fig. 3.

For the artificial cycle operation, oral estradiol valerate 4–6 mg will be given once a day starting from the fourth menstrual cycle day 3 to 5 of the fourth menstrual cycle. When the thickness of the endometrium is > 7 mm on vaginal B ultrasound, patients will be administered with dydrogesterone (10 mg three times per day, orally) and progestin (40 mg daily, intramuscularly). FET will then be performed 3 days after administration of dydrogesterone and progestin. Progesterone will be administered after FET for sustained luteal support until 10 weeks after gestation. But when the endometrial thickness exceeds 15 mm, the FET will be canceled.

### Control group

No intervention will be administered to the control group during the 3 menstrual cycles. However, an artificial cycle will be performed during the fourth menstrual cycle to prepare the endometrium before embryo transfer.

### Adherence

A coordinator in the research center will be in charge of the case report form (CRF) to ensure that data for each participant is accurately recorded. Several methods will be used to explore patient treatment compliance, including frequency of administration of herb-partitioned moxibustion on the navel and weekly telephone follow-up to ensure the findings are valid and reliable. Patients who failed to receive timely treatment on the same day will be identified and their data recorded.

### Concomitant care

Participants will not be allowed to take other Chinese herbal supplements or nutritional supplements to enhance ER for 3 months prior to enrollment into the study as other supplements may affect the herb-partitioned moxibustion applied on the navel.

### Outcome measurements

#### Primary outcomes

The primary outcome is the rate of ongoing pregnancy. It is defined as a detectable fetal heartbeat after 12 weeks of gestation.

#### Secondary outcomes

Secondary outcomes include the following six aspects: (1) endometrial type, (2) RI and PI of bilateral uterine artery and endometrial blood flow, (3) E_2_ and P, (4) biochemical pregnancy rate, (5) implantation rate, and (6) clinical pregnancy rate. The first three secondary outcomes will be compared with baseline in the mid-luteal phase of the third menstrual cycle. The first two secondary outcomes will be measured using transvaginal ultrasound and Doppler after the patient emptied the bladder and rested for 15 min (ultrasound with Siemens G50, probes 4/9 MHz) [29]. Biochemical pregnancy is defined as a positive pregnancy test result (serum β-hCG levels > 10 mIU/mL) 12 to15 days after FET. The implantation rate refers to the number of gestational sacs scanned by transvaginal ultrasound/ the total number of embryos transferred. Clinical pregnancy rate is defined as the presence of a gestational sac on ultrasound with fetal heart tones at 7 weeks of gestation.

#### Retention

The research assistant will conduct weekly follow-ups to remind the participants of upcoming doctor/data collection appointments after enrollment and will record any adverse effects of the herb-partitioned moxibustion on the navel.

### Statistical analysis

#### Sample size calculation

The PASS software version 11.0 (NCSS, LLC. Kaysville, Utah, USA.) was used to estimate the sample size. According to a previous study [30], when the sample size of the treatment group and control group is 94 and 94 respectively, 80% of the power was achieved to detect the assumed rate of ongoing pregnancy in the untreated control group versus the effect size to be examined for (20%). The test statistic used was the two-sided *Z* test with pooled variance and the significance level of the test is 0.05. The significance level achieved in this design was 0.0496. The lost follow-up rate will be estimated by 10% and the final sample size of the treatment group and control group was 105 cases each and the total sample size was 210 cases.

#### Randomization and allocation

Participants will be randomly allocated into treatment or control groups at a rate of 1:1 by a computer-generated randomization sequence generated by the data coordinating center with SAS software version 9.2 (SAS Institute, Cary, NC). Allocation will be made by a designated researcher who will have no contact with any participant and will not be involved in the collection and analysis of the data. Due to the nature of the intervention, it will not be feasible to conceal the allocation from the practitioner and participants. The practitioner will not be allowed to disclose any information concerning their treatment to the participants or the assessor.

#### Data management and monitoring

Three levels of data monitoring will be used to improve quality control. The reproductive medicine practitioner will make the trial and study plan available to all participant, and those who met the inclusion criteria and expressed interest in participating will sign the written informed consent form. The participant’s demographic information will be carefully recorded on the CRF. The supervisors will check the CRF for completeness and accuracy of input data. Any errors will be underlined, corrected, and signed by the corresponding investigator. An independent data monitoring committee (DMC) consisting of an epidemiologist, an acupuncture specialist, and a gynecologist will monitor and evaluate the recruitment of subjects, the progress of research implementation, and safety, confidentiality, and integrity of obtained data monthly. In addition, the DMC will regularly submit audit reports to the project management department. Further, it will provide recommendations for continuing the clinical trial, modifying the trial protocol, or terminating the trial.

#### Data dissemination

The study findings will be disseminated in open access, peer-reviewed journals and shared at international conferences. Research summaries, training tools, manuals, and other related resources were uploaded to the online knowledge management platform.

#### Data analysis

Data will be analyzed using SPSS software version 26.0 (IBM Corp, Armonk, NY). An intention-to-treat analysis (ITT) will be performed to explore the effectiveness of the trial. Patients in the treatment group will be included in the ITT analysis even if they had not completed the herb-partitioned moxibustion on the navel. Continuous variables that will meet the requirements for normal distribution will be represented as mean ± SD. Continuous variables that will not follow normal distribution will be represented as median and IQR. Categorical variables will be reported as absolute and relative percentage frequency. Independent Student *T*-test or Mann-Whitney *U* test will be used to explore differences in mean or median between the treatment group and the control group for continuous variables. Pearson *χ*^2^ test will then be performed to compare baseline characteristics and results for categorical variables. Paired sample *T*-test will be used to compare before and after treatment within-group differences. Multiple imputation will be used to process missing values in the data of this study. All tests will be two-tailed, and the significance level will be set as *P* < 0.05.

#### Protocol amendments

Any modification of the protocol may affect the progress of the clinical study and the potential benefits to participants. If the study objective, study design, patient population, sample size, or study procedure change, the formal revision of the protocol is required. Any amendments to the protocol will be approved by the DMC and submitted to the relevant ethical review bodies.

## Discussion

This is an RCT to evaluate whether the herb-partitioned moxibustion on the navel improves FET outcomes in RIF patients. A total of 210 participants from the Center for External Treatment of Traditional Chinese Medicine and the Reproductive Genetics Center of Integrated Traditional Chinese and Western Medicine in the Affiliated Hospital of Shandong University of Traditional Chinese Medicine will be recruited from March 2021. The results of this study will provide effective evidence that the herb-partitioned moxibustion on the navel improves the pregnancy outcome of women with RIF.

Herb-partitioned moxibustion is a treatment method comprising acupuncture and moxibustion. It exerts combined therapeutic effects of Chinese medicine and moxibustion through Shenque point. The selected traditional Chinese medicines of the herb-partitioned moxibustion on the navel are Loranthus parasiticus, Semen cuscutae, Eucommia ulmoides, Cinnamon, Peach kernel, Red peony root, Angelica sinensis, Ligusticum chuanxiong, Rhizoma cyperi, and Borneol, whose main functions are to tonify the liver and kidney, warm Yang, and promote qi as well as promote blood circulation and remove blood stasis. Related studies [31, 32] found that the endometrial basilar artery RI of patients with RIF decreased after the intervention of Chinese medicine and vaginal ultrasound showed that the echo of the endometrial-myometrial interface (EMI) area was improved and the difference was statistically significant (*P* < 0.05). It showed that Chinese medicines can increase the uterine blood flow perfusion and is helpful to improve patients with ER and early pregnancy outcome. In addition, other studies have confirmed that moxibustion can increase the thickness of the endometrium, improve the blood perfusion in the uterus, and then improve the ER and increase the chance of conception [24, 33]. However, there has been no study on the evaluation of the effects of the herb-partitioned moxibustion on the navel on the outcome of pregnancy in RIF patients. Therefore, the proposed study will administer herb-partitioned moxibustion on the navel of RIF patients to explore the effect on FET outcome.

Despite the lack of convincing evidence, the herb-partitioned moxibustion on the navel is increasingly being used by traditional Chinese physicians to treat infertility. There is an urgent need for a well-designed and well-motivated RCT to demonstrate the efficacy of the herb-partitioned moxibustion on navel in RIF patients and to guide physicians on its use. If the findings show that it is effective, it will provide a new treatment strategy for RIF patients and will promote the use of the herb-partitioned moxibustion on the navel.

## Trial status

The trial was registered at the ChiCTR. This trial is at version 1.5, 8 July 2021 (ChiCTR). The actual study start date was 20 October 2021 and the anticipated study end date is 30 June 2022. The first patient was recruited on July 15, 2021; the anticipated recruitment end date is 31 December 2021.

## Supplementary Information

**Additional file 1.**

**Additional file 2.**

## Data Availability

Datasets generated and analyzed during the study are available from the corresponding author upon request.

## Abbreviations

ER: Endometrial receptivity
RIF: Recurrent implantation failure
IVF-ET: In vitro fertilization and embryo transfer
RCT: Randomized clinical trial
FET: Frozen embryo transfer
PI: Pulsatility index
E_2_: Serum estradiol
P: Serum progesterone
ChiCTR: Chinese Clinical Trial Registry
CRF: Case report form
DMC: Data monitoring committee
ITT: Intention-to-treat analysis
EMI: Endometrial-myometrial interface
