# Supplementary material for: Effectiveness of herb-partitioned moxibustion on the navel for pregnancy outcomes in patients with recurrent implantation failure undergoing in vitro fertilization and embryo transfer: a study protocol for a randomized controlled trial
Source: Trials. 2022 Mar 15;23:217. doi: 10.1186/s13063-022-06156-5 (PMC8922927; doi:10.1186/s13063-022-06156-5)
Supplement: Supplementary file 2 — Additional file 2. [file 13063_2022_6156_MOESM2_ESM.pdf]

山东中医药大学附属医院

中医外治中心、中西医结合生殖与遗传中心

隔药灸脐疗法对反复种植失败患者冻胚移植结局影响的随机对照研究

参与者知情同意书

我们已知反复种植失败患者的妊娠结局不是非常理想。我们仍未了解隔药灸脐疗法能否改善反复种植失败患者的妊娠结局。这项研究的目的是探讨隔药灸脐疗法对反复种植失败患者冻胚移植结局有改善作用。

如果你愿意参与这项研究，你需要：

I. 提供 5-10 毫升血液作生殖内分泌激素或生化检测。抽血的过程中会有很轻微的痛楚，及非常小的感染风险。

II. 花 5-10 分钟的时间填写问卷。

III. 纳入患者随机分入对照组和治疗组。治疗组在胚胎移植前采用隔药灸脐治疗，每周一次，连续治疗三个月经周期，第四个月经周期采用人工周期为冻胚移植作内膜准备；治疗组前三个月经周期不作任何干预，第四个月经周期采用人工周期为冻胚移植作内膜准备。

我们将会在您完成研究后的3个月内致电给您，询问有关妊娠结局的情况。您将授权山东中医药大学附属医院生殖医学伦理委员会查阅有关您涉及研究的资料，作为伦理审查。所有与此项研究有关的资料，均会保密处理。您是否参加此项研究，亦不会影响您应有的治疗。如您对此项研究有任何问题及查询，请致电山东中医药大学附属医院马玉侠教授和宋景艳医师（电话：0531-68617921）。若你对参与此研究的权利有疑问，可于办公时间上午8点至下午5点向山东中医药大学附属医院生殖医学伦理委员会查询（电话：0531-68617980）。

若您现在充分了解有关此项研究的资料而又决定参与，请您在同意书上签名。您的参与全是自愿性质。在签署同意书后，您仍有权随时放弃参与此项研究。您是否参与此项研究， 亦不会影响您应有的治疗。

本人同意参与上述研究。本人明白：

- ✓ 参与此项研究纯属自愿；
- ✓ 本研究不涉及生物样本的获取和保存；
- ✓ 本人有权随时放弃此项研究；
- ✓ 所有资料会保密处理，并只作研究用途；
- ✓ 本人同意*/不同意*与参与研究的机构和监管部门共享临床资料（*请删除其中 1 项）；
- ✓ 若中途退出，本人同意*/不同意*提供临床资料，并将有关数据存储 10 年（*请删除其中 1 项）。

受试者签名：　　　　　　　　　联系电话：　　　　　　　　日期：　　年　　月　　日
研究者签名：　　　　　　　　　联系电话：　　　　　　　　日期：　　年　　月　　日
